# Developing a theory-based health education intervention to prevent adolescent students from smokeless tobacco use

**DOI:** 10.1186/s12889-025-26000-0

**Published:** 2025-12-23

**Authors:** Anil Kumar Mandal, Chitra Bahadur Budhathoki

**Affiliations:** 1 Graduate School of Education, Tribhuvan University, Kirtipur, Nepal; 2 Central Department of Education, Tribhuvan University, Kirtipur, Nepal

**Keywords:** Adolescent, Behavior, Health education intervention, Protection motivation theory, School, Smokeless tobacco, Students

## Abstract

**Background:**

Smokeless tobacco is a kind of tobacco that is chewed or sniffed in the forms of *khaini*, *gutkha*,* pan parag* and *zarda paan with tobacco constituents.* Although these products are harmful to health, over 300 million people worldwide use them. Its use among adolescent students in Nepal is increasing. A theory-based health education intervention is needed to prevent students from consuming smokeless tobacco.

**Method:**

Four focus group discussions were conducted among ninth-grade students following guidelines based on the constructs of protection motivation theory. Based on a rigorous review of the literature and feedback from the supervisor, a module of protection motivation theory-based health education intervention was drafted. Subsequently, a pilot test of the intervention was conducted among the students of grade 9. Data from 16 students who participated in the pilot test were analyzed to identify the reliability and face validity of the intervention. The content validity of the intervention was assured through feedback from the supervisor and the literature. Subsequently, the final draft of the theory-based health education intervention was prepared.

**Results:**

**S**tudents who participated in focus group discussions had insufficient knowledge regarding smokeless tobacco and its harmful effects. They had many misconceptions regarding smokeless tobacco use. They recognized the necessity of acquiring skills to prevent smokeless tobacco use. They voiced that strict rules should be implemented on school premises to control tobacco use. Drawing on such information and findings derived from the needs assessment, a protection motivation theory-based health education intervention was developed. Cronbach’s alpha values of each objective of each session were greater than 0.8. More than 80% of participants in the pilot test agreed with the effectiveness and appropriateness of the components of the intervention.

**Conclusion:**

We developed a highly reliable and valid protection motivation theory-based health education intervention. Its proper implementation might raise students’ knowledge regarding smokeless tobacco and its effects and enhance their skills to prevent its use.

**Supplementary Information:**

The online version contains supplementary material available at 10.1186/s12889-025-26000-0.

## Background

Smokeless tobacco (SLT) is a kind of tobacco that is consumed through the mouth or nose without ignition [1] and is also known as spitting tobacco [2]. There are different types of SLT products such as *khainii* (dried/fermented tobacco leaves with slaked lime), *gutkha (*betel quid with tobacco), *pan parag (pouch containing tobacco constituents with spices and flabours )* and zarda *paan masala (chewing betel leaf quid with tobacco*,* areca nut*,* slaked lime*,* spices and flavour* [2, 3], *tobacco leaf*, *kiwam* [2], *snuff*, and *gul* [3], which are widely consumed in the South-East Asia Region, where 77% of the global SLT users live [4]. SLT contains approximately four thousand toxic, mutagenic, and carcinogenic chemicals, along with highly addictive *Nicotine* [5]. Cancer of the mouth and pharynx [6], heart diseases [7], and gingival inflammation, tooth decay, oral submucous fibrosis, and leukoplakia [8] are attributed to SLT use.

Although SLT use is detrimental to health, over 300 million people in worldwide use SLT [9] and it is attracting more adolescents [10]. Although it has created a compound and pervasive challenge to health and economy, especially in low- and middle-income countries (LMICs) [11], researchers and policymakers have not focused on it, and international efforts of tobacco control are mostly centered on cigarettes rather than other forms of tobacco [9–12]. They neglect it because SLT is considered less harmful than smoking and is a regional problem that is more complicated to control [9]. Similarly, dodging the laws against tobacco and advertising SLT as an alternative to cigarette smoking to maintain nicotine levels by manufacturers of SLT are helpful for smoking cessation programs, but it ultimately increases the prevalence of SLT [10].

In addition, unauthorized and unorganized producers of SLT are mushrooming in Nepal and also a huge amount of SLT is imported from India [3], the world’s largest SLT market with both unorganized/unauthorized and organized/authorized SLT companies [1]. Many large companies in India, such as the Tej Ram Dharam Pal (TRDP) Group (https://trdp.in/), Dharampal Satyapal (DS) Group, and Dilbagh Group, play a significant role in producing and supplying SLT products inside and outside the country. Although the government has prohibited the advertisement and promotion of tobacco products, companies have adopted brand-stretching and surrogate advertisement policies to sell SLT products. Currently, the internet [10] and social media [1] have become means of advertising SLT products, mainly targeting youths [13].

At the global level, many efforts such as proper execution of programs against smoking, parental and peer education, keeping content against tobacco in the curriculum of schools, broad smoke-free policies, and banning tobacco factories are applied to control the use of tobacco among adolescents [14]. Likewise, in Nepal, many initiatives and efforts are being implemented to minimize tobacco use. Nepal signed the World Health Organization Framework Convention on Tobacco Control (WHO FCTC) on December 3, 2003, and ratified it on November 7, 2006 [15]. In addition, the MPOWER policy package was adopted and implemented in 2007 [16] by the government of Nepal to control tobacco use. In the process of meeting the obligations of the WHO FCTC, Nepal has covered more than half of its requirements [17]. Nepal has demonstrated a high level of compliance with advertisement bans, health warnings, and smoking bans in public places, but struggles with mass media, plain packaging, and cessation programs; however, it has achieved partial compliance regarding taxes for tobacco products.

Furthermore, the Government of Nepal has passed the Tobacco Product (Control and Regulatory) Act 2011 [18], which explicitly defines smoking and smokeless tobacco [19]; however, anti-tobacco policies and programs are mainly focused on smoking. Some provisions of the Act prohibit selling tobacco to minorities, selling within 100 m of school premises, using tobacco at public places, and advertising tobacco products, which aims to control and reduce tobacco use. However, selling tobacco products within 100 m of school premises is universal in Kathmandu Metropolitan City [20], which mirrors the situation in the entire country. In addition, the majority of students are not aware of tobacco control policies in Nepal and are not restricted from buying tobacco due to their age [21], indicating that despite well-documented and legislated tobacco control policies in Nepal, its implementation and monitoring system is not effective [22]. In addition, because of the ineffective implementation of adolescent-focused anti-tobacco laws and regulations, the prevalence of tobacco use remains high among them [23].

Although many efforts are being made to control and reduce tobacco use, the prevalence of SLT use among adolescents aged 13–15 years is higher (boys = 4.4%, girls = 2.8%, both = 3.6%) in the South-East Asia Region, except in the Eastern Mediterranean and African Regions of WHO [4]. In Nepal, several initiatives have been implemented to control tobacco use [18, 24, 25], but the prevalence of tobacco use in Nepal remains high. In addition, a report showed that the great majority of people shifted their tobacco use behavior from smoking to SLT use, indicating a higher increment of SLT use in the upcoming days [26]. Likewise, chewing paan with tobacco constituents is the most generalized behavior in the Terai region of Nepal [15], including gutkha and paan masala, which might attract more adolescents to use such products. This suggests the need for evidence-based anti-SLT programs to control and reduce its use among adolescents.

In this regard, a protection motivation theory (PMT)-based health education intervention would be a better alternative to prevent adolescent students from using SLT. PMT emphasizes not only knowledge related to the issue but also preventive measures and the skills needed to cope with such issues [27]. The selected theory is useful for studying intention and concurrent health behaviors of people through threat appraisal and coping appraisal-related variables [28]. PMT is appropriate for studying health behaviors such as tobacco use [29], cigarette smoking, drug abuse, alcohol use, and sexual risk behaviors [30], investigating sustainable waste management behaviors [31], reducing skin cancer risk [32], and understanding sedentary behavior [33] in different contexts. This indicates the broad applicability and implications of PMT-based studies in different settings.

Adolescents are considered more vulnerable to risk-taking behavior [34], and use of tobacco is a door for drugs such as marijuana and cocaine [35, 36] and alcohol. Furthermore, empirical evidence indicates that use of SLT is increasing among youths of Nepal compared to their counterparts in other East Asian countries [37], underscoring the critical need for targeted interventions. These facts indicate that adolescents should be awakened and equipped with knowledge and skills against SLT use. Moreover, PMT provides a suitable theoretical framework for investigating tobacco use behavior among adolescents, although few studies have followed PMT [38]. In this regard, this study aimed to develop a PMT-based health education intervention to prevent adolescent students from consuming SLT.

## Methods

### Study design

The stages of module development include drafting an intervention, identifying its validity and reliability, and finalizing the draft [39]. A needs assessment based on PMT, drafting the intervention module, conducting a pilot test, and finalizing the intervention module were the procedures for developing the health education intervention (HEI). In addition, a rigorous literature review and regular feedback from the supervisor were used to develop the intervention.

### Protection motivation theory (PMT)

In 1975, Rogers developed protection motivation theory (PMT) by including fear appeal variables in expectancy-value theory [40, 41] and modified it in 1983 [27]. Perceptions of severity, vulnerability, intrinsic and extrinsic rewards, response efficacy, self-efficacy, and response cost are the constructs of the modified PMT. Higher perception of severity, vulnerability, response efficacy, and self-efficacy and the lower perception of intrinsic and extrinsic rewards, and response cost raise the protection motivation, which pushes a person to change their attitude and behavior.

### Needs assessment

Students’ needs were assessed through focus group discussions (FGDs) before drafting the intervention module. For this, information-rich students to obtain the rich data for the issue of interest were purposively [42, 43] selected from grade 9 in four rural community secondary schools in the Saptari district to maintain homogeneity and minimize the effects of heterogeneity [44], keeping the objectives of the study in mind. Four to five boys and three to four girls were recruited from each school for each FGD session [45], ensuring the participation of both users and non-users of smokeless tobacco. The students possessed similar backgrounds, and their age ranged from 13 to 16 years. According to the administration and Head Teachers of the selected schools, most students were from lower-and lower-middle-class because poverty is more prevalent in the rural areas than in urban areas [46]. Students who were interested in participating, fell within the age range of 10 to 19 years, and were permanent residents of rural areas were included in the FGD sessions. On the other hand, students who were not regular students, were not in normal health condition, and were residents of urban areas were excluded from the FGDs. We developed guidelines for FGDs (see Additional file 1) based on the constructs of PMT and adhered to them throughout the study. We conducted four FGD sessions [47]—one in each selected school in a separate classroom to prevent disturbances such as noise, hesitation to share their experiences, distraction, and interferences of other students during the discussion. FGDs were conducted with 7–9 students in each session, using both local Maithili and Nepali, and continued until the point of saturation [48]. Each session lasted from 50 min to one hour. A mobile model Vivo 27 was used to record the audio. The audio recordings of the FGDs were transcribed into Nepali. The transcripts were read several times to make sense. Themes were manually extracted from the transcripts of the FGDs by following the sequence of codes, categories, and themes, and then translated into English.

### Drafting of intervention

The intervention module was drafted based on the objectives, guided theory, target group and their needs, and time allocated for the study [39], including insights from literature; regular feedback from my PhD supervisor and an expert of health education, a Professor in Health Education having long experiences in teaching and learning, and researches related to health education; and insights gained from the pilot test and suggestions achieved from students during informal talks.

### Pilot test of intervention

A pilot test of the intervention was conducted to assess its feasibility and identify its reliability and validity. For this, students from grade 9 from a rural community secondary school were purposively selected to ensure similarities and minimize dissimilarities in the background characteristics of the participants [44]. First, the study’s purposes were briefly described to the students, and all of them were invited to participate in the pilot study. However, altogether 25 students participated in the pilot test, only the data of 16 students (boys-7, girls-9), aged 14 to 16 years, were included in the analysis because students who missed at least one session were excluded. Among the excluded students, seven missed one session, and two missed two sessions. Although a larger size is needed for better precision during the pilot test [49], the included samples were appropriate to demonstrate the ability to execute a protocol and test acceptability of adherence of the intervention for the feasibility study [50, 51], determine its face validity [51, 52], and provide insights to modify the number of sessions based on the time taken to complete it. The researchers conducted a pilot test of the HEI, two sessions each week, among the selected ninth-grade students. Each session took 45 min to complete, except for the first and the concluding sessions. Each of the first session and the concluding session had a duration of 90 min. Thus, the pilot test was completed in four weeks, including one extra concluding session in the fourth week. The pilot test of the intervention was conducted according to the school’s schedule in a separate classroom; however, the timing of the sessions was adjusted each week to minimize disruption to other classes.

### Reliability of intervention

A questionnaire based on the objectives of the module was used for identifying its reliability (Jamaludin Ahmad and Sidek Mohd Noah, 2001) [as cited in 39]. We developed and used a self-reported questionnaire based on the objectives of each session—except the concluding session—to calculate Cronbach’s alpha and identify the reliability of the intervention (see Additional file 2). The questionnaire contained 72 items, three for each of the 24 objectives of the eight sessions. The questionnaire used a five-point Likert scale type, ranging from strongly disagree (1) to strongly agree (5). After one week of completing the pilot test of the intervention, data were collected by administering questionnaires to the students. The researchers facilitated guidance on any confusion or difficulties related to the questions. After data collection, the data were edited and entered into the Statistical Package for Social Science (SPSS) version 20 to calculate Cronbach’s alpha to ensure the reliability of the intervention module.

### Validity of intervention

Content validity was ensured through a comprehensive review of the literature and consultation with experts [53]. The participants of the pilot test, a subgroup of the study population [48], were used to ensure the face validity of the intervention contents. We developed a self-reported five-point Likert-scale-type questionnaire ranging from strongly disagree (1) to strongly agree (5) along with the questionnaire for the reliability test of the intervention (see Additional file 2), and administered it among the students to identify the face validity of the intervention module. It contained seven items related to contents, activities, teaching materials, and time allocated for the sessions. However, it was analyzed by categorizing it into disagree, neutral, and agree. Likewise, informal 3- to 5-minute talks with three students about the contents and activities of the intervention modules were conducted to ensure their face validity.

### Logic model of HEI

**Fig. 1:**
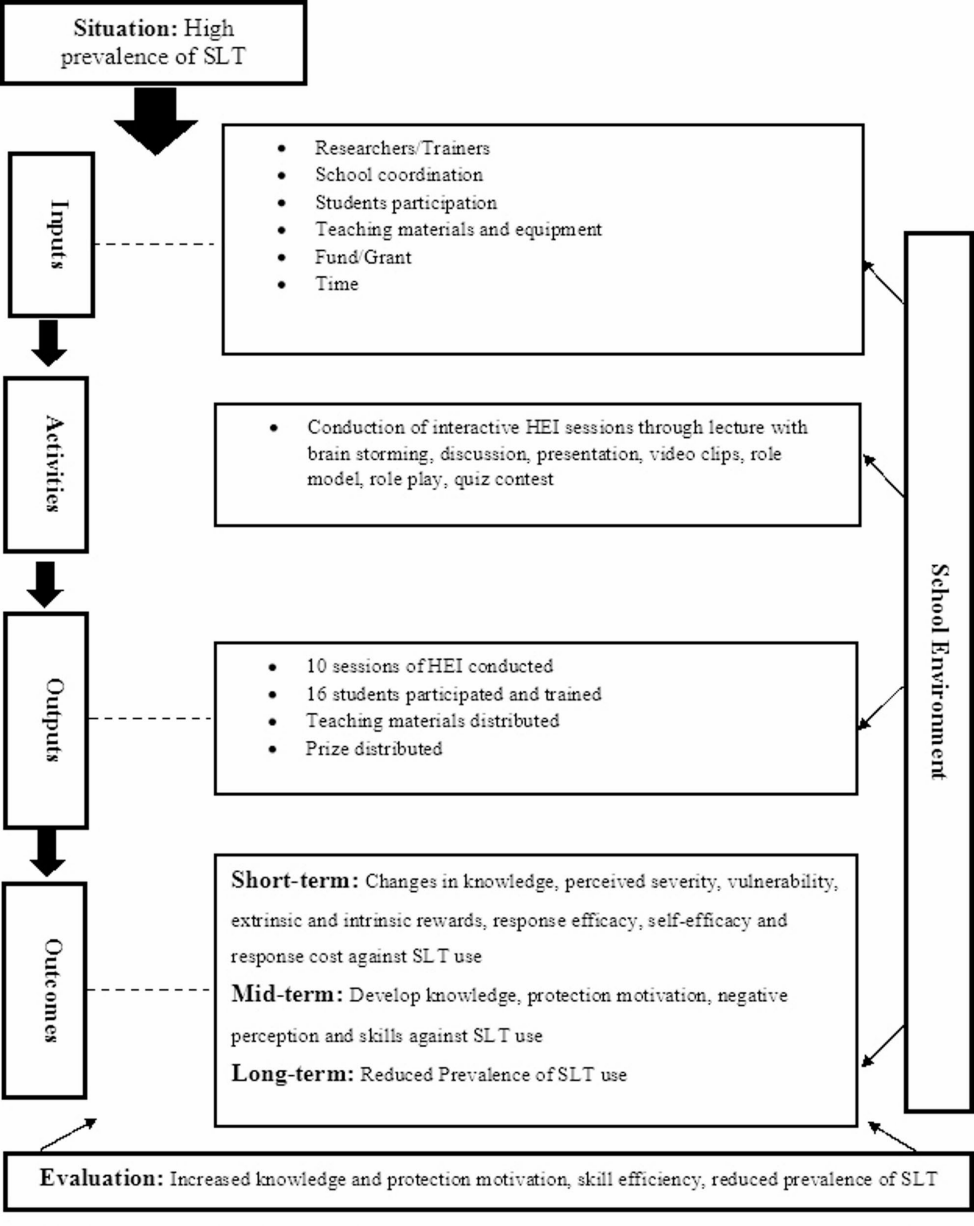
Logic model of HEI

The HEI logic model (Fig. 1) provides important information about the components of the intervention, such as situations, environments, inputs, activities, outputs, outcomes and evaluation criteria. Considering the high prevalence rate of SLT use among adolescent students, an HEI was planned. Researchers/trainers, schools, students, teaching materials and equipment, funds/grants, and time allocated for the interventions were the inputs of HEI. The HEI sessions primarily involved different interactive teaching and learning methods, including a quiz contest. The number of conducted sessions, the number of participants and trained students, the distributed teaching materials, and the prizes were the HEI outputs. Changes in the level of knowledge, perceived severity, vulnerability, extrinsic and intrinsic rewards, response efficacy, self-efficacy and response cost against SLT use after the HEI would be the short-term outcome of the HEI. Developing and solidifying knowledge, protection motivation and skills against SLT use following short-term outcomes would be a mid-term outcome. Finally, the reduced prevalence of SLT use among adolescent students following mid-term outcome would be the long-term outcome of the HEI. Evaluation was planned to be conducted on the basis of participants’ level of knowledge, protection motivation, skill efficiency and prevalence of SLT use among adolescent students before and after the HEI.

## Results

A draft module of the theory-based health education intervention was developed based on the themes derived from FGDs data, which was aligned with constructs of PMT. The themes derived from FGDs were knowledge related to SLT, effects of SLT use (severity of SLT), hazards and addiction of SLT use (vulnerability of SLT), misconceptions related to rewards of SLT use (intrinsic and extrinsic rewards), preventive measures of SLT use (response efficacy), skills to prevent SLT use (self-efficacy), misconceptions related to the cost of not consuming SLT (response cost), and peers as the source of SLT use.

### Knowledge related to SLT

The study revealed that students had insufficient knowledge about SLT and its dangerous effects. They described *‘it is food like food habit (nasha) for many individuals’* (FGD 4) in response to what they know about tobacco. They were unable to differentiate between tobacco and alcohol. They thought that tobacco, alcohol, and drugs were the same thing. One of the participants explained *‘cigarette*,* bidi* (hand-rolled smoked tobacco product), *alcohol*,* gaza* (cannabis/marijuana), *water gun*,* amrit plus*,* rajniwash*,* bhola*,* rajanigandha as different types of tobacco products’* (FGD 1). Similarly, they were unaware of the hazardous chemicals found in SLT. One student said that *‘they do not know about the chemicals found in it’*. They showed some normative education regarding tobacco use. They further assumed that ‘*over 90% of youths and adults consume tobacco’* (FGD 3).

### Effects of SLT use

Students had some knowledge of the effects of SLT use. One of the students in FGD said that *‘chewing it*,* is not good*,* chewing tobacco is not good’* (FGD 1). They only mentioned *‘harm to the body*,* and weakening the body’* in response to what its effects are. Another student replied, *‘…cancer may occur*,* use of it causes cancer of the neck’*. The next student responded that it damages the lungs. Another student claimed that ‘*it may damage kidneys*’ (FGD 4). In addition, they replied, *‘it disturbs the mind*,* disturbs people and the mind becomes weak’*. However, some students knew the immediate effects of SLT use. Some students pointed out that *‘…teeth and lip become black and the eyes of people who consume become red’* (FGD 2).

### Hazards and addiction of SLT use

Students had some ideas about potential risks associated with SLT use. They were aware of the chemical responsible for addiction, but they did not know more about it. Although they lacked sufficient knowledge about the hazardous chemical found in SLT, they mentioned that it contained certain chemicals that were responsible for addiction. They accepted that *‘if you get addicted once*,* it is more difficult to quit’* (FGD 1). Another student said *‘…get addicted and then consume again and again’* (FGD 4). People might suffer from diseases due to tobacco use. In addition, its use might cause death. Accepting this fact, one student stated that ‘*death might occur due to use of it’* (FGD 3).

### Misconceptions related to the rewards of SLT use

Misconceptions related to the rewards of SLT use are one of the major reasons for using it. In support of this, one student said, *‘it gives good taste at first use…’* Another student added, *‘after consuming*,* I feel relaxed*,* do not ask me more’* (FGD 1). People believe that individuals use tobacco to reduce tension. Regarding this, one student replied, *‘It means those who experienced some problems such as dizziness and mental irritation may begin to use it’* (FGD 3). Participants in FGD sessions echoed that *‘yes*,* people consume while they are in tension’*. Many people associate SLT use with prestige and social status. One of them further stated, *‘After consuming*,* say that I am like this*,* I am like that; and some people consume it to show off’* (FGD 4).

### Preventive measures of SLT Use

Students agreed and suggested different preventive measures for SLT use. One student voiced that *‘selling of tobacco should be banned’*. One of them continued, *‘A strict rule should be made and enforced*’ (FGD 4) to control SLT use. They emphasized the need for an awareness program to control tobacco use. They echoed that *‘raise voice against tobacco use*,* like a voice raised for dowry by going village to village’* (FGD 1) to control tobacco use. Another student added that *‘checking should be done every day*,* and an additional teacher should be appointed*,* that PT teacher…’* (FGD 1) are ways to prevent tobacco use. Regarding alternative healthy behavior, they echoed that *‘it might be quitted by chewing lwang* (clove) *and sauf* (fennel seeds), *which have no nasha (addiction)’* (FGD 2).

### Skills to prevent SLT use

Almost all students accepted the need for skills like decision-making, coping with peer pressure and saying ‘no’ to a friend’s proposal of SLT use. Students had some idea about how to refuse friends’ proposals, but they did not have an appropriate idea about decision-making and coping with peer pressure. One student said, *‘I convince them not to consume it*,* it is not good’* (FGD 4) in response to a friend’s proposal of SLT use. One student voiced that *‘I tell them whether I am with you at each step*,* but I do not consume*,* they can consume’* (FGD 2).

### Misconceptions related to the cost paid for not consuming SLT

Students had misconceptions regarding the costs of not using SLT. They believe that they may face many consequences if they do not consume SLT. One student said, *‘The person who consumes it says that I may not work*,* I may not be alive if I do not consume’*. Sometimes, friends create pressure on their friends to consume it in many ways. One of the participants said, *‘…Addicted friends usually propose other friends to use SLT. If they declined to use it*,* they warned that if they did not use it*,* then they would have to leave the gang*,* live separately. You are not our friend you don’t accept our proposal’* (FGD 1).

### Peer as the source of SLT use

Peers are the source and motivator of SLT use. Regarding this, one student said that *‘…if any friends consume*,* then they others’*. They continued, *‘…When someone is consuming and others have then*,* they tell them to consume it*,* and so consuming once may attract them to consume again and again’* (FGD 1). Sometimes, peers create pressure on their friends to consume tobacco. One student expressed that *‘if not consume give on hands and forced to consume’* (FGD 2). Repeated proposals by friends are one of the reasons of SLT use. To support this, one student said, *‘After proposing one or two times*,* then give*,* I consume’*.

We found that many students did not have sufficient knowledge of SLT. They considered tobacco, alcohol, and drugs as forms of tobacco. They believed that smoked and smokeless tobacco were the same. They were unaware of the harmful chemicals found in SLT and their effects. They believed that almost all people consume tobacco, and more people who do not consume tobacco are suffering from cancer and dying than those who consume tobacco. Some of them also thought that not consuming SLT is good for health. They said that consuming SLT is relaxing and prestigious among people. They accepted that peer pressure was a dominating factor in consuming SLT. They recognized the need for life skills to prevent SLT use. They echoed the need for an awareness program and the implementation of strict rules in school settings to prevent tobacco use.

### Drafting and finalization of intervention

Before the pilot test, HEI had nine sessions, including one concluding session. For each of the seven PMT constructs, one session of intervention was drafted, except for self-efficacy, which had two sessions.

After the pilot test, we slightly modified HEI. The 1st session was divided into two sessions, the third and fourth sessions were merged into one session, and the seventh session was divided into two sessions. It had 10 sessions without altering the contents and objectives, which it had before the pilot test, including a quiz contest and a concluding session. Session 1st had focused on knowledge related to SLT. Session 2nd was related to the severity of SLT use, session 3rd was related to the vulnerability of SLT, session 4th was related to the extrinsic and intrinsic rewards of SLT, session 5th was related to response cost, session 6th was related with response efficacy, and sessions 7th, 8th, and 9th were concerned with self-efficacy. Thus, contents related to each of the seven PMT constructs was included in the intervention. The 10th session was a quiz contest and concluding session, which was related to the contents of sessions 1 through 9 to solidify the knowledge and skills acquired by students in each session. Table 1 presents the final number of sessions, along with the contents and objectives of the intervention after the pilot test.

**Table 1:** Sessions of the intervention with contents and objectives

Sessions	Contents	Objectives
1. Introduction to smokeless tobacco (SLT)	- Introduction - Types of SLT - Chemicals found in SLT	- introduce SLT - identify different types of SLT - list chemical found in SLT
2. Prevalence and effects of SLT use	- Prevalence of SLT - Short term effects of SLT - Long term effects of SLT - Mortality caused by tobacco	- explain prevalence of SLT use - describe short terms effects of SLT - describe long terms effects of SLT - describe mortality caused by tobacco
3. Addiction and hazards of SLT use	- Addiction of SLT - Probability of hazards caused by SLT use - Positive aspects of no SLT use	- explain the process of addiction due to SLT use - describe the probability of hazards caused by SLT use - list the positive aspects of not consuming SLT
4. Misconception related to rewards of SLT Use	- Misconceptions related to Intrinsic rewards of SLT use - Misconceptions related to intrinsic rewards of SLT use	- identify misconceptions related to intrinsic rewards of SLT use - explain misconceptions related to extrinsic rewards of SLT use
5. Misconceptions related to cost paid for no SLT use	- Misconceptions related to cost paid for no SLT use - Social/Group norms	- describe misconceptions related to cost paid for not consuming SLT - able to differentiate positive and negative group norms
6. Preventive measures of SLT use	- Preventive measures of SLT	- explain preventive measures of SLT use - accept the importance of prescribed behaviors to prevent from SLT use
7. Decision making	- Introduction of decision making - Steps of decision making	- describe decision making - list the steps of decision making - able to apply the steps of decision making in their behavior
8. Peer pressure	- Introduction of peer pressure - Ways of coping peer pressure	- describe peer pressure - explain the ways of coping peer pressure - able to apply the ways of coping peer pressure in behavior
9. Saying ‘NO’ SLT	- Introduction of saying ‘NO’ SLT - Different ways of saying ‘NO’ SLT	- describe different ways of saying ‘NO’ SLT - able to apply the different ways of saying ‘NO’ in their behavior
10. Quiz contest and conclusion	- Quiz related to above sessions	- to solidify the knowledge and skill achieved in the previous sessions

### Reliability of intervention

The Cronbach’s alpha values of each objective of the respective sessions were high and acceptable, ranging from 0.807 to 0.880. The Cronbach’s alpha values for each objective are presented in Table 2.

**Table 2:** Objective-wise Cronbach alpha of each session

Sessions	No. of objectives for each session	No. of questions for each objectives	Value of Cronbach Alpha
1. Introduction to Smokeless Tobacco (SLT)	1	3	0.824
	2	3	0.826
	3	3	0.857
2. Prevalence and Effects of SLT Use	1	3	0.880
	2	3	0.842
	3	3	0.815
	4	3	0.815
3. Addiction and Hazards of SLT Use	1	3	0.824
	2	3	0.828
	3	3	0.864
4. Misconceptions Related to Rewards of SLT Use	1	3	0.822
	2	3	0.865
5. Misconceptions Related to Cost Paid for not Consuming SLT	1	3	0.867
	2	3	0.807
6. Preventive Measures of SLT Use	1	3	0.827
	2	3	0.814
7. Decision Making	1	3	0.817
	2	3	0.858
	3	3	0.818
8. Peer Pressure	1	3	0.836
	2	3	0.821
	3	3	0.838
9. Saying ‘NO’ SLT	1	3	0.846
	2	3	0.846

### Validity of intervention

More than 80% of students agreed on the effectiveness and appropriateness of all components, except the time allocated for each session. Less than 70% of students agreed with the time allocated for each session. During informal conversations with students, they mainly suggested breaking down the first session into two sessions and merging the third and fourth sessions into one session. Agreement percentages by participants for each component of the sessions are presented in Table 3.

**Table 3:** *Agreement percentages for each component of the sessions*

Components	D (%)	A (%)
The contents were clear and understandable	12.5	87.5
The contents were useful to meet the needs of students	6.2	93.8
The contents meet the learning standard of students	12.5	87.5
The learning materials are clear and readable	12.5	87.5
The teaching-learning activities were appropriate to meet the needs of students	12.5	87.5
The teaching-learning activities created the opportunity for active learning	12.5	87.5
The time allocated for each session was appropriate	31.2	68.8

*D* Disagree, *A* Agree

## Discussion

Inadequate knowledge regarding tobacco use may lead to undesired attitudes [54]. But, we found that students had insufficient knowledge of tobacco use, including SLT, and its adverse health effects. Only a few of them knew that the chemicals contained in SLT are responsible for addiction and diseases like cancer. In addition, they were misinformed about the probability of health hazards of SLT use, misconceptions about rewards related to SLT use, and misconceptions related to the cost of not consuming SLT. Therefore, information related to it was included in the HEI sessions.

Including the contents that are related to different aspects of SLT in the intervention might have a positive impact on the SLT use behavior of students because knowledge about the harms of tobacco is one of the protective factors of its use [55]. In addition, Wang et al. [56] reported that the school-based tobacco use prevention program ‘towards no tobacco use (TNT)’ was a more highly cost-effective intervention than other prevention programs. Similarly, Tahil et al. [57] argue that school-based smoking interventions raise knowledge of the negative impacts of smoking and enhance a negative attitude towards smoking among students. Similarly, Reed et al. [58] assessed the implementation of a spit tobacco prevention curriculum in West Virginia and found a positive impact on students’ knowledge of tobacco use. Therefore, we can say that providing knowledge about SLT might increase students’ knowledge.

Misconceptions regarding the rewards of SLT use and the cost of not consuming SLT are significant factors in its use. Considering smoking as attractive and convenient at social activities is a significant factor of smoking [59]. People consume tobacco as a stress reducer, and some people consume tobacco to maintain body weight [60] and to look fit and smarter. However, a study [61] showed that very few students perceived tobacco use as a cool, comfortable enhancer and stress reducer; some people accept that tobacco consumers had more friends. Likewise, personal attitude towards tobacco and social norms are significant contributors to tobacco use [62]. In the FGDs, students voiced such misconceptions related to SLT use; therefore, information about such misconceptions was included in the intervention.

Skills are essential for applying the knowledge acquired and the attitudes developed. Social cognitive theory [63] emphasizes the need and importance of self-efficacy in performing actions. It accepts the perception of self-efficacy as a vital component for mastering the desired action or behavior. Maddux and Rogers [40] emphasized self-efficacy as one of the main constructs of PMT for achieving the desired behavior. However, the FGDs showed that participants had insufficient skills related to decision-making, coping with peer pressure, and saying ‘NO’ to their peers’ proposals for SLT use. They felt hesitant regarding the skills necessary to keep themselves away from SLT use. All of them accepted the need for skills to prevent SLT use. Keeping this in mind, some life skills such as decision-making, coping with peer pressure, and saying ‘NO’ to SLT were included in the HEI sessions. This intervention may have a positive impact on the smoking cessation behavior of students, as evidenced by the study conducted in West Virginia by Reed et al. [58], who reported that implementing the curriculum had a positive effect on students’ tobacco use behavior. They mentioned that almost all students were able to list the things to purchase instead of spit tobacco, to say no to their friends who offered spit tobacco, and to get more involved in the prevention of spit tobacco use in their school or community.

Participants of FGDs accepted peers as the main source of SLT use. Therefore, contents related to peer pressure and how to refuse a proposal of a peer to consume SLT were included in the intervention. Many studies have demonstrated the effectiveness of peer-oriented intervention. Lund et al. [64] emphasized that prevention programs should focus on the role of peers and family to control it. Similarly, a peer-led, school-based tobacco control intervention had a significant effect on reducing SLT use [65].

Adolescence (10–19 years) is a transitional period between the stages of childhood and adulthood, and adolescents might be involved in risk behaviors [66] and lack the capacity to make the right decision. During this period, guidelines for making the right decisions are required. In the absence of decision-making capability, they might be involved in risks such as SLT use, substance abuse, and violence rather than positive and creative works. The capability of decision-making skills is an asset of youths and a symbol of positive identity and social competencies that lead them to constructive and positive behavior [67]. Therefore, it is necessary to enhance their decision-making capability to prevent them from risk-taking behaviors such as the use of SLT.

Researchers have no consistency regarding the value of Cronbach’s Alpha to set the standard of reliability of an intervention. Good and acceptable values of Cronbach’s Alpha varied from 0.60 to 0.80 [53] and from 0.65 to 0.90 [39]. However, we found a high and acceptable reliability for the intervention because no sessions had a Cronbach’s Alpha value of less than 0.8.

We obtained a high face validity for the components of the intervention sessions. Almost all students agreed with the contents and learning activities included in the intervention sessions. However, some (one-third) students disagreed with the time allocated for the session, especially the first session, which was modified. It requires an agreement percentage of 75 to be valid, and an agreement percentage below 75 needs to be revised [68]. In this regard, the first session was divided into two sessions, the third and fourth sessions were merged into one session, and the three sessions were separated for self-efficacy to manage time.

This study had some limitations. The needs assessment was limited to the PMT constructs. In the intervention, we included only decision-making, managing peer pressure, and refusing the proposal of SLT use across various life skills. It had limited sessions and a time duration. The pilot test of the intervention was limited to a small number of students from a rural community secondary school. The use of a self-reported questionnaire to collect data was another limitation of this study, which may have created the possibility of response bias. However, the intervention module (HEI) mainly focuses on raising the knowledge and protection motivation of students against SLT. In addition, it clarifies misconceptions related to SLT use and enables them to decide not to use SLT, cope with peer pressure, and refuse friends’ proposals of SLT use. Therefore, it might be adapted to adolescent students in different settings and Southeast Asian countries, where the prevalence of SLT use is high. In addition, it would add a brick to the literature and to the field of tobacco control programs. This would add a new dimension to tobacco control programs by developing a protection motivation theory-based, smokeless tobacco-specific health education intervention to control and prevent smokeless tobacco use. This would serve to initiate and provide guidelines for designing school-level health education curricula by including smokeless tobacco-specific content in the curriculum.

## Conclusion

We developed a highly reliable and valid PMT-based health education intervention based on the students’ needs. Finally, it had ten sessions, including a quiz contest and a concluding session, whereas it had nine sessions during the pilot test. Each session took forty-five minutes to complete, except for the quiz contest and concluding session. It can be anticipated that proper implementation of this intervention would enhance students’ knowledge, protection motivation, and skills regarding SLT prevention and assist them in applying these in their lives. Therefore, local bodies and school stakeholders should include this intervention in their policies and programs.

## Supplementary Information

Supplementary Material 1.

Supplementary Material 2.

## Data Availability

The data used for the current study will be available from the corresponding author on reasonable request.

## Abbreviations

FCTC: Framework Convention on Tobacco Control
PhD: Philosophy of Doctor
PMT: Protection Motivation Theory
SLT: Smokeless Tobacco
UGC: University Grant Commission
WHO: World Health Organization
