# Supplementary material for: Developing a theory-based health education intervention to prevent adolescent students from smokeless tobacco use
Source: BMC Public Health. 2025 Dec 23;26:328. doi: 10.1186/s12889-025-26000-0 (PMC12837446; doi:10.1186/s12889-025-26000-0)
Supplement: Supplementary file 1 — Supplementary Material 1. [file 12889_2025_26000_MOESM1_ESM.pdf]

Guidelines for Focus Group Discussion

Needs assessment for developing Health Education Intervention

Time: Place: Date:

Facilitator/Interviewer:

Participants/Interviewee:

[Brief introduction of study- purpose, process, ethical concerns]

- Briefly describe what tobacco/smokeless tobacco is. (Introduction, different types of smokeless tobacco, prevalence of smokeless tobacco)
- Which chemicals are found in smokeless tobacco? (Addictive, Cancerous)
- What are the effects of smokeless tobacco consumption? (Short-term effects, long-term effects, mortality attributed to tobacco use)
- What are the probable hazards of smokeless tobacco consumption? (Being addicted, being sick, early death, benefit of not consuming smokeless tobacco)
- What types of rewards do students think they achieve from smokeless tobacco consumption? (Intrinsic rewards such as feeling of pleasure, reduction of tension, having mood fresh; Extrinsic rewards such as having more friends, looking smart, increase social relationship)
- What do people have to pay if they refuse peer proposals of smokeless tobacco consumption? (If not consumed- forced to leave group, being angry, abusing, threatening)
- How would smokeless tobacco consumption among students be prevented? (awareness programs, rule-regulations, alternative healthy behaviors)
- Why are life skills necessary to prevent students from smokeless tobacco consumption? (Decision-making, saying ‘No’ to peer proposals of smokeless tobacco, managing peer pressure)

Thank you!
