# Supplementary material for: Developing a theory-based health education intervention to prevent adolescent students from smokeless tobacco use
Source: BMC Public Health. 2025 Dec 23;26:328. doi: 10.1186/s12889-025-26000-0 (PMC12837446; doi:10.1186/s12889-025-26000-0)
Supplement: Supplementary file 2 — Supplementary Material 2. [file 12889_2025_26000_MOESM2_ESM.pdf]

Questionnaire to Assess Reliability of Health Education Intervention

Sex: **Age:** **Class:**

To what extent do you agree or disagree with the following statements? Please, circle any one of the numbers based on your experiences during the sessions of intervention.

[Strongly disagree (SD) = 1; Disagree (D) = 2; Neutral (N) = 3; Agree (A) = 4; Strongly agree (SA) = 5]

S. No.	Statements	SD	D	N	A	SA
1.	I can introduce SLT.	1	2	3	4	5
2.	I can define SLT.	1	2	3	4	5
3.	I can describe SLT.	1	2	3	4	5
4.	I can classify different types of SLT products.	1	2	3	4	5
5.	I can explain different types of SLT products.	1	2	3	4	5
6.	I can give examples of different types of SLT.	1	2	3	4	5
7.	I can list name of chemicals found in SLT.	1	2	3	4	5
8.	I can explain chemicals found in SLT.	1	2	3	4	5
9.	I can tell the names of most cancerous chemicals found in SLT.	1	2	3	4	5
10.	I can describe the prevalence of SLT consumption in the world.	1	2	3	4	5
11.	I can explain the prevalence of SLT consumption among adolescents.	1	2	3	4	5
12.	I can share facts related to the prevalence of SLT consumption with others.	1	2	3	4	5
13.	I can describe short-term effects of SLT consumption.	1	2	3	4	5
14.	I can list short-term effects of SLT consumption.	1	2	3	4	5
15.	I can share short-term effects of SLT consumption with others.	1	2	3	4	5
16.	I can describe long-term effects of SLT consumption.	1	2	3	4	5
17.	I can list long-term effects of SLT consumption.	1	2	3	4	5
18.	I can share long-term effects of SLT consumption with others.	1	2	3	4	5

S. No.	Statements	SD	D	N	A	SA
19.	I can describe the proportion of mortality attributed to tobacco use.	1	2	3	4	5
20.	I can explain the mortality caused by tobacco consumption in a year.	1	2	3	4	5
21.	I can share the facts related to the proportion of mortality attributed to tobacco consumption with others.	1	2	3	4	5
22.	I can tell the name of chemical responsible for addiction of SLT.	1	2	3	4	5
23.	I can describe how the consumption of SLT leads to its addiction.	1	2	3	4	5
24.	I can share why we get addicted to SLT with others.	1	2	3	4	5
25.	I can describe probable hazards of SLT consumption.	1	2	3	4	5
26.	I can list probable hazards of SLT consumption.	1	2	3	4	5
27.	I can share probable hazards of SLT consumption with others.	1	2	3	4	5
28.	I can describe positive aspects of not consuming SLT.	1	2	3	4	5
29.	I can list positive aspects of not consuming SLT.	1	2	3	4	5
30.	I can share positive aspects of not consuming SLT with others.	1	2	3	4	5
31.	I can describe misconceptions related to intrinsic rewards of SLT consumption.	1	2	3	4	5
32.	I can list misconceptions related to intrinsic rewards of SLT consumption.	1	2	3	4	5
33.	I can share misconceptions related to intrinsic rewards of SLT consumption with others.	1	2	3	4	5
34.	I can describe misconceptions related to extrinsic rewards of SLT consumption.	1	2	3	4	5
35.	I can list misconceptions related to extrinsic rewards of SLT consumption.	1	2	3	4	5
36.	I can share misconceptions related to extrinsic rewards of SLT consumption with others.	1	2	3	4	5

S. No.	Statements	SD	D	N	A	SA
37.	I can describe misconceptions related to the cost paid for not consuming SLT.	1	2	3	4	5
38.	I can list misconceptions related to the cost paid for not consuming SLT.	1	2	3	4	5
39.	I can share misconceptions related to the cost paid for not consuming SLT with others.	1	2	3	4	5
40.	I can describe positive and negative norms related to SLT consumption.	1	2	3	4	5
41.	I can differentiate positive and negative norms related to SLT consumption.	1	2	3	4	5
42.	I can share positive and negative norms related to SLT consumption with others.	1	2	3	4	5
43.	I can describe preventing measures of SLT Consumption.	1	2	3	4	5
44.	I can list preventive measures of SLT consumption.	1	2	3	4	5
45.	I can share preventive measures of SLT consumption with others.	1	2	3	4	5
46.	I can list prescribed behaviors that prevent SLT consumption.	1	2	3	4	5
47.	I am able to describe the importance of prescribed behaviors that prevent SLT consumption.	1	2	3	4	5
48.	I can share prescribed behaviors that prevent SLT consumption with others.	1	2	3	4	5
49.	I can introduce decision-making.	1	2	3	4	5
50.	I can define what decision-making is.	1	2	3	4	5
51.	I can explain decision-making, linking it with SLT consumption.	1	2	3	4	5
52.	I can describe the steps of decision-making.	1	2	3	4	5
53.	I can list the steps of decision-making.	1	2	3	4	5

S. No.	Statements	SD	D	N	A	SA
54.	I can explain the steps of decision-making regarding SLT consumption.	1	2	3	4	5
55.	I am able to act upon the steps of decision-making.	1	2	3	4	5
56.	I am able to demonstrate the steps of decision-making regarding not consuming SLT.	1	2	3	4	5
57.	I am able to apply the steps of decision-making regarding not consuming SLT in my life.	1	2	3	4	5
58.	I can describe peer pressure.	1	2	3	4	5
59.	I can give examples of peer pressure related to SLT consumption.	1	2	3	4	5
60.	I can share what is peer pressure with others.	1	2	3	4	5
61.	I can describe ways of managing peer pressure.	1	2	3	4	5
62.	I can explain ways of managing peer pressure, linking it with SLT consumption.	1	2	3	4	5
63.	I can share ways of managing peer pressure with others.	1	2	3	4	5
64.	I am able to demonstrate how to manage measures of peer pressure.	1	2	3	4	5
65.	I am able to manage peer pressure regarding SLT consumption.	1	2	3	4	5
66.	I am able to act upon steps of managing peer pressure.	1	2	3	4	5
67.	I can describe ways of saying "No" to peer proposals of SLT consumption.	1	2	3	4	5
68.	I can list different types of saying "No" to peer proposals of SLT consumption.	1	2	3	4	5
69.	I can explain different types of saying "No" to peer proposals of SLT consumption.	1	2	3	4	5
70.	I am able to demonstrate how to say "No" to peer proposals of SLT consumption.	1	2	3	4	5
71.	I am able to say "No" to peer proposals of SLT consumption.	1	2	3	4	5

S. No.	Statements	SD	D	N	A	SA
72.	I am able to apply different types of saying "No" to peer proposals of SLT consumption in my life.	1	2	3	4	5
**Questionnaire to Assess Face Validity of Health Education Intervention**						
1.	The contents of the sessions were clear and understandable.	1	2	3	4	5
2.	The contents of the sessions were useful to meet the needs of students.	1	2	3	4	5
3.	The contents of the sessions met the learning standard of students.	1	2	3	4	5
4.	The teaching materials used in the sessions were clear and readable.	1	2	3	4	5
5.	The teaching-learning activities of the sessions were appropriate to meet the needs of students.	1	2	3	4	5
6.	The teaching-learning activities of the sessions created the opportunity for active learning.	1	2	3	4	5
7.	The time allocated for each session was appropriate.	1	2	3	4	5

Thank you!
